# A generation of junior faculty is at risk from the impacts of COVID-19

**DOI:** 10.1371/journal.pbio.3001266

**Published:** 2021-05-25

**Authors:** Tiffany M. Lowe-Power, Louise Dyson, Abigail M. Polter

**Affiliations:** 1 Department of Plant Pathology, University of California, Davis, California, United States of America; 2 The Zeeman Institute for Systems Biology & Infectious Disease Epidemiology Research, School of Life Sciences and Mathematics Institute, University of Warwick, Coventry, United Kingdom; 3 Department of Pharmacology and Physiology, George Washington University, Washington, DC, United States of America

## Abstract

For junior investigators starting their independent careers, the challenges of the COVID-19 pandemic extend beyond lost time and are career threatening. This Perspective article argues that without intervention, academic science could lose a generation of talent.

## Teaser

For junior investigators starting their independent careers, the challenges of the Coronavirus Disease 2019 (COVID-19) pandemic extend beyond lost time and are career threatening. Without intervention, academic science could lose a generation of talent.

For academic scientists, the past year has been a period of lost time and slow progress. This time has been exceptionally stressful for investigators in the early stages of their career, such as postdoctoral researchers, graduate students, and junior faculty. For pretenure faculty, the challenges of Coronavirus Disease 2019 (COVID-19) are occurring at a particularly critical juncture and threaten the long-term stability of their careers. The career progression of junior PIs hinges on their ability to turn the early resources invested in them into meaningful scientific results. For this cohort of investigators, a significant portion of their start-up funds and first grants have been used to fund salaries of staff who were unable to work at full capacity and wasted resources (research animals, labile reagents, and access to field sites). The use of these resources without progress toward scientific goals will be devastating to the careers of researchers who lack long track records of independent productivity to fall back on. The pandemic has also generated logistical challenges, including supply shortages, restrictive immigration policies, and institutional hiring and spending freezes. These hardships disproportionately hamper the work of junior investigators who are trying to stock and staff their new labs. Travel restrictions and social distancing policies have limited opportunities for both local and global networking and collaboration. These restrictions are detrimental to junior faculty who face the challenge of establishing a national reputation in order to be granted tenure. This confluence of challenges has stalled momentum for junior faculty at a time when it is crucial for their research programs to grow.

It is important to note that within the category of junior faculty, the negative effects of the pandemic have not been equally distributed. Women, especially those who have family care responsibilities, have been particularly affected [1]. Additionally, preexisting structural racism and sexism [2] may have limited resources and support networks available for underrepresented groups, leaving them with less structural resilience to weather the disruption of the pandemic. Furthermore, junior faculty are not the only group whose careers are threatened by the pandemic; many of these same hardships also impact early career researchers such as postdoctoral fellows who have not yet obtained an independent position. These researchers will also need concentrated efforts to ensure that their careers can be maintained past the pandemic.

How can funding bodies and academic institutions best prevent the loss of early career faculty due to the pandemic? The most pressing need is for financial reinvestment. This is absolutely essential if junior faculty are to regain momentum. Funding bodies should offer targeted funding aimed at stabilizing the financial situation of junior PIs and giving them the resources needed to “catch up” from the pandemic. This could take the form of short-term bridge awards, such as an expansion of the NIH R56 and similar programs for those who have not yet received funding, or of funded extensions for those who recently received their first major award. Expansion of funding for longer-term new investigator awards such as NSF CAREER awards would also be helpful. Furthermore, funding bodies should issue blanket extensions of eligibility for time-limited career development awards and early stage investigator status. Academic institutions should also help maintain the careers of junior faculty by providing resources, such as augmentation of start-up funds, availability of bridge funding, and enhanced in-kind support. Institutions can also ease financial difficulties for new faculty by providing increased flexibility for spending existing start-up funds and extending deadlines by which startup funds must be used. We are cognizant that funding bodies and academic institutions are working with limited budgets, thus it will be critical for scientists and administrators to advocate to the government and their institutions that increased research funding for junior investigators is critical to recovering from the pandemic and ensuring that the investments already made in junior faculty are not lost.

Junior faculty also face lost time and momentum. Many universities responded to the pandemic with rapid assurances of tenure extensions. These extensions can be helpful, but they are insufficient to fully address the harms of the pandemic and have drawbacks for faculty who take them. Delaying tenure carries personal financial impacts and prolongs the stress of the precarious tenure track. Extensions to the tenure clock are not always interpreted equitably; for example, fathers’ careers benefit from taking a tenure extension while mothers’ do not [3]. Institutions will need to work carefully to ensure that tenure extensions are equitable to all faculty that take them. A helpful alternative or addition to tenure extensions would be to give junior faculty a semester or year of teaching and service release, allowing them to focus exclusively on restarting their research program.

As the COVID-19 pandemic fades into memory, it is essential that the senior scientists making career-defining decisions on hiring, tenure, and funding for junior academics do not forget the impact the pandemic has had. The financial and logistical effects of the pandemic should be documented as much as is possible, in annual reviews and mid-tenure checkpoints. It is important that institutional tenure and promotion committees develop holistic and transparent review guidelines for evaluating pandemic-affected academics and that they carefully monitor success rates of attaining tenure for this cohort. We recommend that tenure dossiers should include a statement from the candidate and their chair documenting the specific effects of the pandemic on their lab and that this document be seriously discussed as part of deliberations on each case.

COVID-19 has shifted the career trajectories of this generation of early career academics. Decisions about hiring, funding, tenure, and promotion should be made based on how well an individual is doing on this new trajectory, not whether they have been able to claw their way back to the pre-COVID standard. Hiring and tenure committees must be prepared to adjust their expectations and make their evaluations based on what candidates have achieved with the time and resources available to them. Importantly, “adjusted expectations” do not mean “lowered expectations.” This generation of early career academics are surviving an unprecedented disaster while maintaining research programs, adapting their pedagogy to teaching online, and preserving safety of lab members and their families. Juggling these responsibilities demonstrates ingenuity, tenacity, hard work, and empathic leadership. Junior academics have done this with limited resources while in a career phase of immense stress, uncertainty, and pressure. To persist and succeed despite the pandemic demonstrates that junior faculty are valuable members of their institutions and scientific community. Tenure committees must recognize that junior academics did not spend the pandemic doing less work, but doing different, equally important work. Junior faculty represent the future of our fields, but the impacts of the pandemic have imperiled their career progression. It will take significant efforts from the scientific community to ensure that this generation of scientists is not a casualty of the pandemic.
